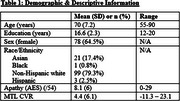# Apathy and medial temporal lobe cerebrovascular reactivity

**DOI:** 10.1002/alz70856_107043

**Published:** 2026-01-11

**Authors:** Jillian L. Joyce, Arunima Kapoor, Megan S Barker, John Paul M Alitin, Allison C Engstrom, Trevor Lohman, Fatemah Shenasa, Aimée Gaubert, Farrah Ferrer, Amy Nguyen, Xingfeng Shao, Danny JJ Wang, Daniel A. Nation

**Affiliations:** ^1^ University of Southern California, Los Angeles, CA, USA; ^2^ University of California, Irvine, Irvine, CA, USA; ^3^ Brown University, Providence, RI, USA; ^4^ Leonard Davis School of Gerontology, University of Southern California, Los Angeles, CA, USA; ^5^ University of Southern California, Leonard Davis School of Gerontology, Los Angeles, CA, USA

## Abstract

**Background:**

Apathy is a common neuropsychiatric symptom in Alzheimer's disease (AD) and cerebral small vessel disease, and is characterized by a pathological lack of motivation or interest in activities. It is often evident in the preclinical stages of disease, and individuals with apathy experience faster functional declines and poorer quality of life. Despite its clinical significance, the biological mechanisms underlying apathy are not well understood. Apathy has been associated with white matter hyperintensities of presumed vascular origin, warranting further investigation into vascular contributions to apathy. Cerebrovascular reactivity (CVR) provides a dynamic and functional measure of vascular health. CVR quantifies how effectively cerebral blood vessels modulate blood flow in response to vasoactive stimuli (i.e. CO2 increase during breath‐hold). CVR deficits within the medial temporal lobe (MTL) are associated with poorer memory performance and greater risk of dementia. However, no prior work has investigated the association between apathy and CVR. In this study, we examined the association between CVR in the MTL and apathy in older individuals without dementia.

**Method:**

This study included 121 community‐dwelling older adults without dementia or stroke. Participants completed brain MRI and the Apathy Evaluation Scale (AES). Pseudo‐continuous arterial spin labeling MRI quantified cerebral perfusion during breath‐hold induced vasodilation (three 15‐second visually guided breath holds) in the MTL (hippocampus and parahippocampal gyrus). Lower CVR values indicate reduced vasodilatory abilities and poorer vascular function. Linear regressions evaluated the relation between CVR and AES, with age as a covariate.

**Result:**

Lower CVR abilities associated with greater apathy endorsement (*β* = ‐.250, *p* = .006). Age did not associate with CVR (*β* = ‐.045, *p* = .614).

**Conclusion:**

These results highlight the link between vascular dysfunction and apathy among older adults. Impaired CVR in the MTL may be indicative of vascular dysfunction in this region, which could disrupt brain networks critical for goal‐directed behavior, motivation, and emotion. Elucidating vascular contributions to apathy could inform interventions that improve functional outcomes and quality of life.